# Ascertaining glacier dynamics and geodetic mass changes in the Pangong Region of Trans-Himalayan Ladakh using remote sensing data

**DOI:** 10.1016/j.dib.2022.108176

**Published:** 2022-04-12

**Authors:** Irfan Rashid, Nadeem Ahmad Najar, Ulfat Majeed, Waseem Rasool

**Affiliations:** Department of Geoinformatics, University of Kashmir, Hazratbal Srinagar, Jammu and Kashmir 190006, India

**Keywords:** Glacier recession, Remote sensing, Geodetic mass changes, Glacier inventory, Ladakh Himalaya

## Abstract

Glaciers in the Himalayan arc are receding rapidly in the eastern and western parts as compared to other regions. Contrararily, the glaciers in the Trans-Himalayan region of Ladakh are comparatively stable. The differential retreat could be due to various climatic, topographic, and geologic influences. The use of multi-source remotely sensed imagery from open-source platforms and the GlabTop model has been discussed in this paper. This paper draws insights from a recently published paper which details the recession of 87 glaciers in the Trans Himalayan region of Ladakh using remote sensing data [1]. The use of remote sensing data from USGS and Planet Labs for assessing glacier area changes, frontal retreat, debris cover, topographic characteristics, and comparison with existing inventories has been discussed in this study. The geodetic mass changes have been assessed using SRTM and TanDEM-X of 2000 and 2012 respectively. The use of remotely sensed data discussed in this article will help glaciologists to better characterize and understand the glacier recession in the region. The GlabTop model has been used to simulate proglacial lake expansion to understand glacier-bed overdeepenings of four glaciers in the region. The GlabTop simulations will help disaster managers to better quantify the vulnerability and risk of downstream population and infrastructure to Glacial Lake Outburst Floods (GLOFs).

## Specifications Table

**Table:** 

Subject	Earth and Planetary Sciences
Specific subject area	Glaciology; Glacier Recession; Glacier-related disasters
Type of data	Image and Vector, TIFF and SHP files
Data Acquisition	Moderate-resolution Landsat TM and SRTM datasets were downloaded from USGS Earth Explorer (http://www.earthexplorer.usgs.gov/). High-resolution satellite data was procured from Planet Labs (http://www.planet.com). TanDEM-X was procured from (https://tandemx-science.dlr.de/). Glacier outlines that comprise Randolph Glacier Inventory (RGI), Glacier Area Mapping for Discharge from the Asian Mountains (GAMDAM) inventory, and ICIMOD were downloaded from https://www.glims.org/RGI/rgi-60_dl.html, http://www.cryoscience.net/gamdam20180404.zip, and rds.icimod.org/Home/DataDetail?metadataId=9375&searchlist=true respectively. The GlabTop model was provided by Dr. Andreas Linsbauer from the University of Zurich.
Data format	Raw
Description of data collection	Glacier area changes and frontal retreat were assessed using satellite data. Glacier surface lowering was ascertained using multidate DEMs. Proglacial lake expansion was estimated using GlabTop model. Topographic characteristics were determined from TanDEM-X. Debris cover was manually delineated using high-resolution Planet Cubesat images. Glacier inventories were compared in a GIS environment.
Data source location	Name of the study area: Pangong City/Town/Region: Trans-Himalaya, Ladakh Country: India Latitude: 33°36′−34°2′N Longitude:78°14′−78°39′E Mean elevation: 5110 m asl
Data Acessibility	Repository name: Mendeley Data Data identification number: 10.17632/y4hph4cr22.3 Direct link to the dataset: https://data.mendeley.com/datasets/y4hph4cr22/3
Related research article	U. Majeed, I. Rashid, N.A. Najar, N. Gul, Spatiotemporal dynamics and geodetic mass changes of glaciers with varying debris cover in the Pangong Region of Trans-Himalayan Ladakh, India between 1990 and 2019, Front. Earth Sci. 9 (2021) 748,107 https://doi.org/10.3389/feart.2021.748107.

## Value of the Data

• The data about spatiotemporal glacier changes, topography, debris cover, geodetic mass changes and glacier ice thickness can contribute towards an improved understanding of the glacier dynamics and proglacial lake evolution in the Trans-Himalayan Ladakh where in situ data are not available.
• The data can benefit policy makers and water resource managers for sustainable management of water resources in Ladakh region. The data would be immensely helpful to disaster management authorities like National Disaster Management Authority (NDMA, India), State Disaster Management Authority (SDMA) and State Disaster Response Force (SDRF) for GLOF risk reduction to communities and infrastructure downstream.
• The data generated in this study could serve as a baseline for any detailed glaciological (field-based mass balance, debris depth characterization, proglacial lake bathymetry) and environmental (environmental impact assessment, anthropogenic footprint, environment planning) investigations in the rapidly developing Pangong Region.

## 1 Data Description

This section describes the multisource datasets that were used to quantify the glacier recession, frontal retreat, physiographic characterization of glaciers, glacier-wise geodetic mass changes, potential glacier bed overdeepening vis-à-vis proglacial lake evolution, and comparison of existing glacier inventories in the Pangong Region of Trans-Himalayan Ladakh, India. Landsat TM images (Spatial resolution: 30 m) of 1990 and Planet Cubesat images (Spatial resolution: 3 m) of 2019 helped quantify the area changes and frontal retreat of the Pangong group of glaciers. The data about the glacier outlines of 1990 and 2019 are provided in the Mendeley data repository (outlines.rar). An important aspect of the study was to assess the robustness of existing inventories of glacier outlines by comparing them with the manually-delineated glacier outlines in this study. This can help glaciologists in ascertaining the bias associated with the glacier cover and glacier count in the existing global and regional inventories. The data about glacier outlines is available as comparison.rar and comparison.docx. The data about topographic parameters (mean glacier elevation, mean glacier slope, frontal slope of the glacier,% southerly aspect) and debris cover that influence glacier recession [2] is provided in the Mendeley data repository (characterization.rar). The multidate Digital Elevation Models (DEM) of 2000 and 2012 provide important insights into geodetic glacier mass changes [3,4]. The glacier surface lowering data of the Pangong group of glaciers is available as dem.rar in the data repository. GlabTop model was used to simulate glacier bed overdeepenings and glacier ice thickness for the four glacier associated with proglacial lakes [5], [6], [7]. The model results about bed-overdeepenings and ice thickness are available as glabtop.rar. The glacier-wise data is also available as an MS Word file (assessment.docx) in the Mendeley data repository.

## 2 Experimental Design, Materials and Methods

The 87 Glaciers in the Pangong Region were digitized manually at 1:30,000 scale on 1990 and 2019 images to assess the changes in area, snout, and glacial lakes. The debris cover of 2019 was also manually delineated from the Planet CubeSat images of 2019. The standard geometric correction [8] was used to co-register the images. The topographic characterization that includes mean glacier slope, mean glacier elevation, and percentage of southerly aspect were estimated using TanDEM-X in a GIS environment. The geodetic mass changes over the Pangong Region were quantified using DEM differencing of TanDEM-X (2012) from SRTM (2000). A correction factor of 3.4 m for SRTM C band penetration in glacier ice was used [9]. The glacier-bed overdeepenings and ice thickness were calculated for four glaciers associated with proglacial lakes using the GlabTop model to predict the future proglacial lake expansion in the Pangong Region.

## Ethics Statements

No human or animal studies are presented in the manuscript. The study does not involve any data that was acquired from any social media platforms.

## CRediT authorship contribution statement

**Irfan Rashid:** Conceptualization, Methodology, Formal analysis, Investigation, Data curation, Resources, Writing – original draft, Writing – review & editing, Supervision, Project administration. **Nadeem Ahmad Najar:** Conceptualization, Methodology, Software, Validation, Formal analysis, Investigation, Data curation, Writing – original draft. **Ulfat Majeed:** Methodology, Software, Formal analysis, Investigation, Data curation, Writing – original draft. **Waseem Rasool:** Formal analysis, Investigation, Data curation.

## Declaration of Competing Interest

The authors declare that they have no known competing financial interests or personal relationships that could have appeared to influence the work reported in this paper.

## Data Availability

Data for: Ascertaining glacier dynamics and geodetic mass changes in the Pangong Region of Trans-Himalayan Ladakh using remote sensing data (Original data) (Mendeley Data).
